# The Design, Fabrication and Characterization of a Transparent Atom Chip

**DOI:** 10.3390/s140610292

**Published:** 2014-06-11

**Authors:** Ho-Chiao Chuang, Chia-Shiuan Huang, Hung-Pin Chen, Chi-Sheng Huang, Yu-Hsin Lin

**Affiliations:** 1 Department of Mechanical Engineering, National Taipei University of Technology, Taipei 10608, Taiwan; E-Mail: daynight780422@yahoo.com.tw; 2 Vacuum Technology and Nanofabrication Division, Instrument Technology Research Center, National Applied Research Laboratories, Hsin-Chu 30076, Taiwan; E-Mails: chbin@itrc.narl.org.tw (H.-P.C.); yhlin@itrc.narl.org.tw (Y.-H.L.); 3 Opto-Electro-Mechanical System Division, Instrument Technology Research Center, National Applied Research Laboratories, Hsin-Chu 30076, Taiwan; E-Mail: chisheng@itrc.narl.org.tw

**Keywords:** glass substrate, transparent atom chip, heat dissipation

## Abstract

This study describes the design and fabrication of transparent atom chips for atomic physics experiments. A fabrication process was developed to define the wire patterns on a transparent glass substrate to create the desired magnetic field for atom trapping experiments. An area on the chip was reserved for the optical access, so that the laser light can penetrate directly through the glass substrate for the laser cooling process. Furthermore, since the thermal conductivity of the glass substrate is poorer than other common materials for atom chip substrate, for example silicon, silicon carbide, aluminum nitride. Thus, heat dissipation copper blocks are designed on the front and back of the glass substrate to improve the electrical current conduction. The testing results showed that a maximum burnout current of 2 A was measured from the wire pattern (with a width of 100 μm and a height of 20 μm) without any heat dissipation design and it can increase to 2.5 A with a heat dissipation design on the front side of the atom chips. Therefore, heat dissipation copper blocks were designed and fabricated on the back of the glass substrate just under the wire patterns which increases the maximum burnout current to 4.5 A. Moreover, a maximum burnout current of 6 A was achieved when the entire backside glass substrate was recessed and a thicker copper block was electroplated, which meets most requirements of atomic physics experiments.

## 1. Introduction

In 1995, after Cornell and Wieman [1] successfully achieved the Bose-Einstein Condensation (BEC), it has become the research focus in field of quantum optics and atomic physics. Atom trapping chips have also been applied in BEC and atomic physics experiments [2–12]. Wire patterns on atom chips are mainly fabricated by using the lithography and electroplating technology, and a sufficient magnetic field gradient can be generated by applying several amperes of electrical current through them to trap atoms. The atom chip technique dramatically simplifies the experimental effort that is necessary to obtain a BEC. It eliminates high-power, water-cooled magnetic coils and large power supplies. Farkas *et al.* [13] directly miniaturized the vacuum chamber to create a portable BEC vacuum system. In addition, the traditional metal coil capable of running high current can be replaced by atom chips which only require several amperes of current. Therefore, atom chips are widely used in atomic physics and BEC experiments. Previous atom chips were mostly fabricated on silicon substrates and the wire patterns were made of gold. Armijo *et al.* [14] used higher thermal conductivity aluminum nitride (AlN) as a substrate. Some atom chips are designed to reflect laser beams on the chip surface. Chuang *et al.* [15] reported a multilayer atom chip which is fabricated on a silicon substrate with electroplated copper wires on the top surface and a copper thin film is evaporated on the outside area of the electroplated copper wires to reflect the laser beams. In addition, a silver mirror is attached on the chip surface for the purpose of reflecting laser light [16] in order to achieve the laser cooling process. The disadvantages of this method are: (1) the incident angle of the laser light is short of one degree of freedom, (2) the glue used to attach the silver mirror is easily degraded after a period of time, and (3) the deposited copper thin film causes varying reflectivity on different areas of the atom chip due to the different height of the wire patterns [15]. An alternative way is to deposit a gold thin film directly on the chip surface for reflecting the laser beams.

Huet *et al.* investigated some properties of the transparent atom chip made of silicon carbide (SiC) and gold wires [17]. Their results show that a better thermal behavior is observed in the presence of electrical current, twice as good as for a silicon substrate, which makes the single crystal SiC a candidate substrate for atom chip applications. Salim *et al.* [18] proposed a compound silicon and glass atom chip used in a high-resolution projection and imaging system for ultracold atoms experiments. Most areas of this atom chip are metalized to enable the magnetic trapping while coupled specific glass regions provide the optical access to atoms residing in the magnetic trap.

This study presents a transparent atom chip made of a glass substrate, which allows the laser light to penetrate directly through the glass substrate to achieve the laser cooling process to completely solve the problem of laser light reflection. The glass substrate is easily accessible, inexpensive and has a good optical access although it is a poor heat conductor which makes the heat generated from the metal wires on atom chips unable to effectively dissipate. Thus, the maximum electrical current applied through the metal wires decreases. Therefore, in this study, we provide another approach to make a transparent atom chip based on a glass substrate by implementing different heat dissipation methods to enhance the maximum electrical current applied on the metal wires. In addition, an anti-reflection coating was also used to improve the laser light transmission though the glass substrate (see Section 2.5). The concept of the first heat dissipation design on atom chip is to individually fabricate heat dissipation copper blocks around the wire patterns on the front side of the atom chips and reserve an area for the laser light transmitting region. The fabrication process and the electrical current measurements are introduced in Sections 2.2 and 3.3, respectively. In the second and third design, the heat dissipation copper blocks are fabricated right under the wire patterns on the backside of the atom chips to effectively dissipate the heat generated by the wires. The fabrication process and the electrical current measurements are later introduced in Sections 2.3, 2.4, 3.3 and 3.4, respectively. After fabricating transparent atom chips with the heat dissipation design, an E-gun evaporator was used to coat an anti-reflection thin film on both sides of the laser light transmitting region to increase the transmittance of laser light and to achieve the requirements of atomic physics experiments. Hence, the proposed fabrication process for making transparent atom chips can provide a wider application for chip-based atom trapping experiments.

**Figure 1.**
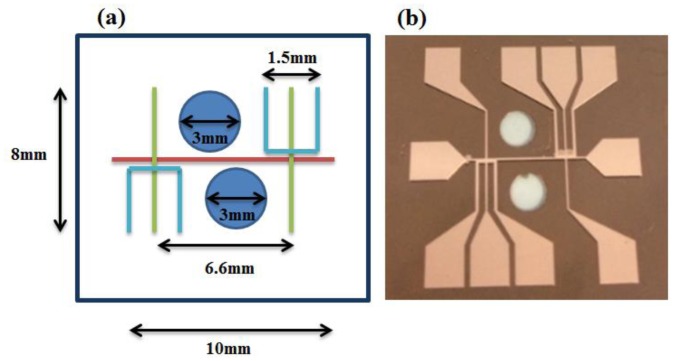
(**a**) Schematic of a final chosen transparent atom chip design with a combination of U and H-wire trap pattern design. (**b**) Photograph of a fabricated transparent atom chip.

In typical atom trapping experiments, the wire pattern designs of atom chips are commonly U, H, Z-type, and generally combined with a uniform external bias field to provide a three-dimensional constraint [19–21]. The magnetic field types and gradients generated by applying current through the wires are different according to various wire shapes. The wire shapes used in this study are those referred to by Chuang *et al.* [15], namely double layer atom chips, except for two laser light transmitting regions with a diameter of 3 mm that are inserted in-between the wire patterns as shown in Figure 1. The wire shapes on atom chips are mainly combinations of U and H-wire trap patterns.

## 2. Fabrication Process

### 2.1. Fabrication of Transparent Atom Chips without Heat Dissipation Design

**Figure 2.**
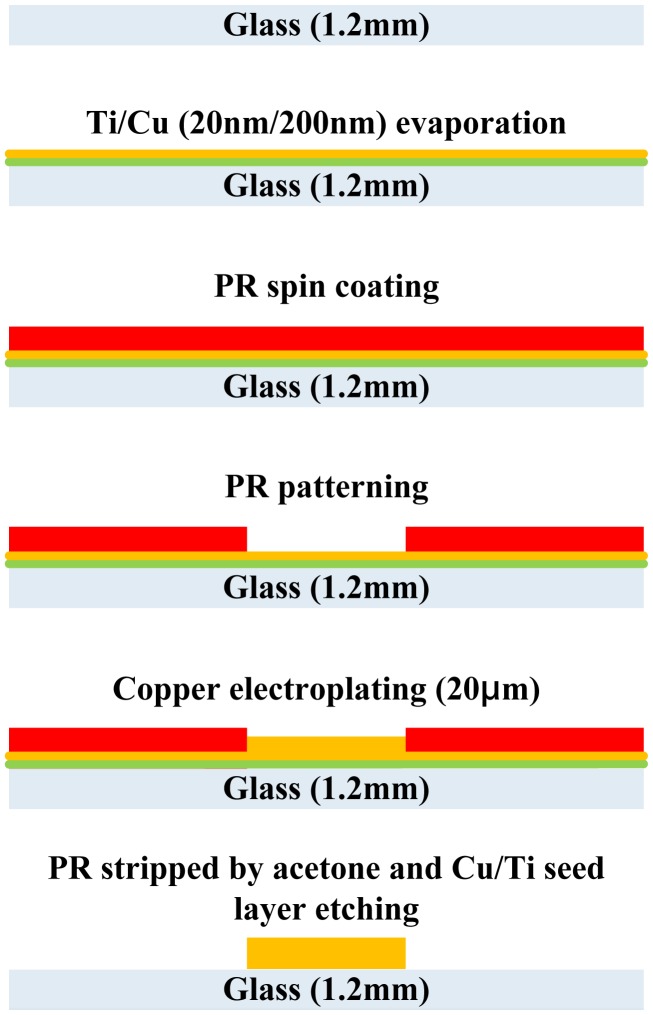
Fabrication process for creating a single layer transparent atom chip without heat dissipation design.

**Figure 3.**
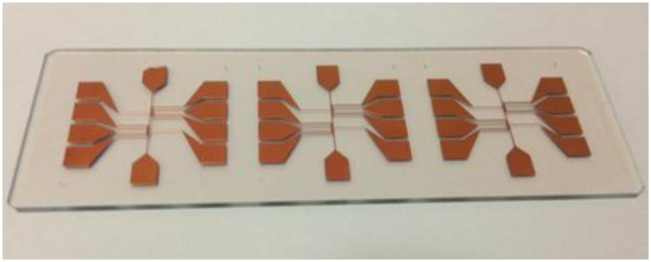
Photograph of three fabricated signal layer transparent atom chips.

The fabrication process of a single layer atom chip without heat dissipation design is shown in Figure 2. An adhesion layer titanium (20 nm) and a seed layer copper (200 nm) are evaporated on one side of the glass substrate by an E-beam evaporation system. A 20 μm thick copper layer is patterned and plated by the general lithography and electroplating process, respectively. The seed layer copper and adhesion layer titanium are etched away by wet etching to isolate the bottom wires. The copper etchant consists of 500 mL DI water, 5 mL sulfuric acid and 60 g Microetch 85 powder from Technic, Inc. (Cranston, RI, USA). The etching rate is approximately 0.25 μm/min at room temperature. The titanium etchant consists of 40 parts DI water, 1 part nitric acid and 1 part hydrofluoric acid. Such etchants have a high chemical etching selectivity and a lower etching rate allowing more control of the etching time. The photograph of the single layer transparent atom chip without heat dissipation design is shown in Figure 3.

### 2.2. Fabrication of Transparent Atom Chips with Heat Dissipation Design on the Front Side

**Figure 4.**
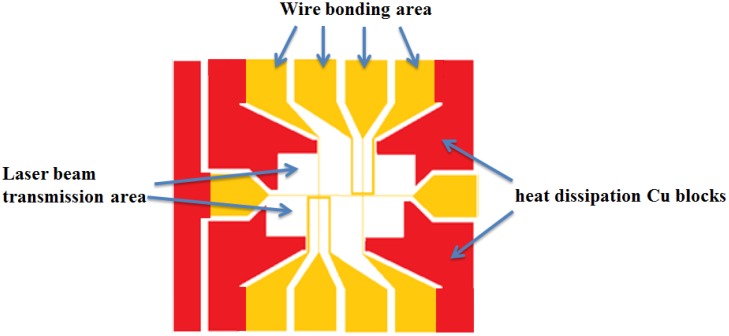
The design of the wires and heat dissipation copper blocks on the mask.

Since the glass substrate has very poor thermal conductivity, the wires on these atom chips can only sustain a maximum burnout current of 2 A without a heat dissipation design, which doesn't meet most requirements of an atomic physics experiment. Thus, the heat dissipation copper blocks design is added around the wire patterns on the front side of the atom chips as shown in Figure 4. The heat generated by the metal wires can be directly conducted to the copper blocks on the front side of the atom chips for dissipation and some certain areas on atom chips are retained as the laser light transmitting region.

### 2.3. Fabrication of Transparent Atom Chips with Heat Dissipation Design on the Backside (Blind Hole)

**Figure 5.**
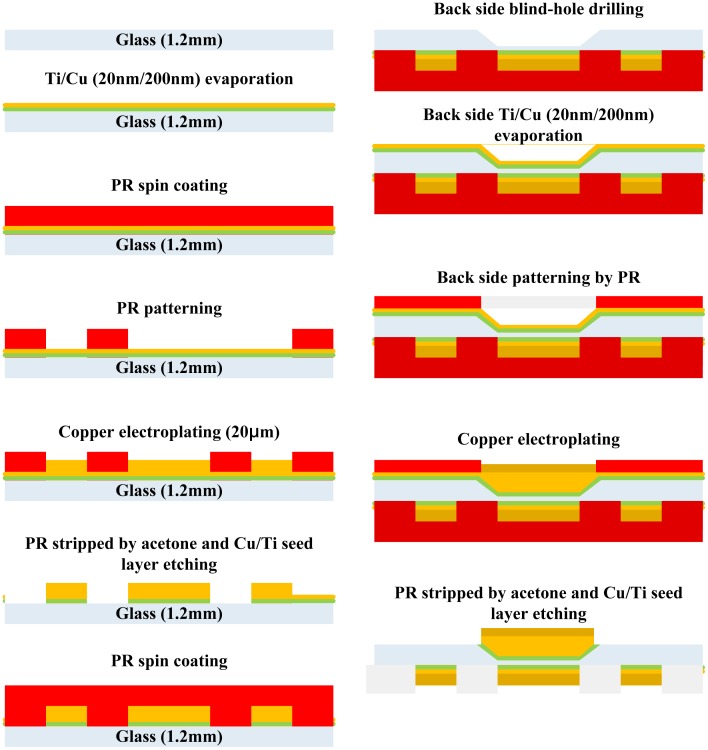
Fabrication process for creating a single layer transparent atom chip with heat dissipation design on the backside (blind-hole).

Four tapered blind holes were drilled from the backside of the glass substrate right below the front side wires to reduce the total thickness of the glass substrate for the purpose of effectively conducting the heat generated by the wires to the backside of the substrate. The heat dissipation copper blocks were then electroplated inside the blind holes and the fabrication process is shown in Figure 5. The glass substrate was cleaned first and the wires were patterned and electroplated on the front side of the atom chip. Four blind holes were then drilled on the backside of the substrate with a tapered diamond drill bit. The backside of the glass substrate was placed into an E-gun evaporation system to deposit the adhesion layer titanium (20 nm) and the seed layer copper (200 nm). The blind holes were patterned on backside of the chips and filled up with the electroplated heat dissipation copper blocks. Finally, the titanium and copper layers were etched away at the laser light transmitting region.

### 2.4. Fabrication of Transparent Atom Chips with Heat Dissipation Design on the Backside (Backside Etching)

The results of the electrical current test from the previous design (see Section 3.3) show that the heat generated from wires can effectively be conducted to the heat dissipation copper blocks (blind holes) on the backside of the substrate, which increases the maximum electrical current that can be applied through the wires. However, due to glass being a brittle material, the glass substrate easily cracks or breaks during the drilling process, causing reduction of the fabrication yield. Thus, the design of the heat dissipation on atom chips is altered. From the previous heat dissipation design (blind holes), it is clearly seen that the thermal conductivity of the glass is very poor. However, if the thickness of the glass substrate is reduced, the heat generated by the wire can still be efficiently conducted to the heat dissipation copper blocks on the backside of the transparent atom chips.

**Figure 6.**
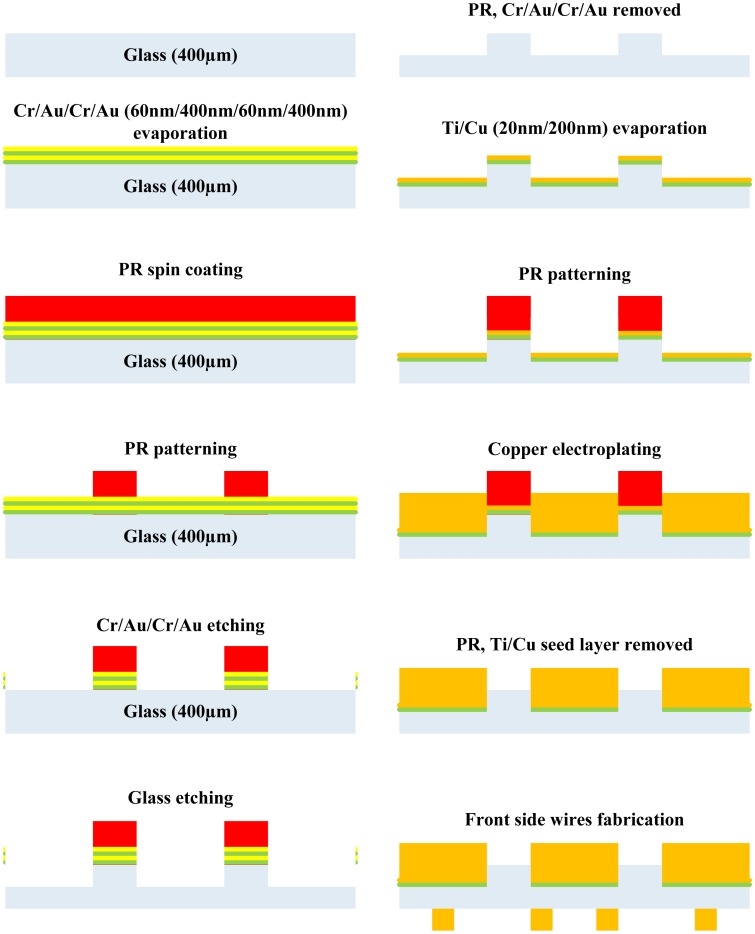
Fabrication process for creating a single layer transparent atom chip with heat dissipation design on the backside (backside etching).

Therefore, in this study, a large area on the backside of the glass substrate was etched away by a hydrofluoric acid (HF) with a concentration of 49%, so that the backside of the glass substrate underneath the wires (front side) can be effectively thinned. During the etching of the glass substrate, the laser light transmitting regions were protected as well and the fabrication process is shown in Figure 6.

The glass (BK7) with a thickness of 400 μm was used as the substrate for fabricating transparent atom chips. The surface of the glass substrate was first cleaned by a standard procedure and then placed into an evaporation system. Since gold and chromium is used as a masking layer for HF etching and the adhesion layer between gold and glass substrate, respectively, multilayers of gold and chromium (Cr/Au/Cr/Au, 60 nm/400 nm/60 nm/400 nm) were evaporated on the glass substrate by the E-gun evaporation system to protect the laser light transmitting regions. After spin-coating the photoresist on the substrate, the HF etching areas can be patterned with a standard lithography process. After hard-baking the photoresist, the masking layer gold and the adhesion layer chromium on the HF etching areas can be etched away by the *aqua regia* (nitric acid: HCl = 1:3) and chromium etchant (CR-7). A large area on the backside of the glass substrate was then etched by HF.

**Figure 7.**
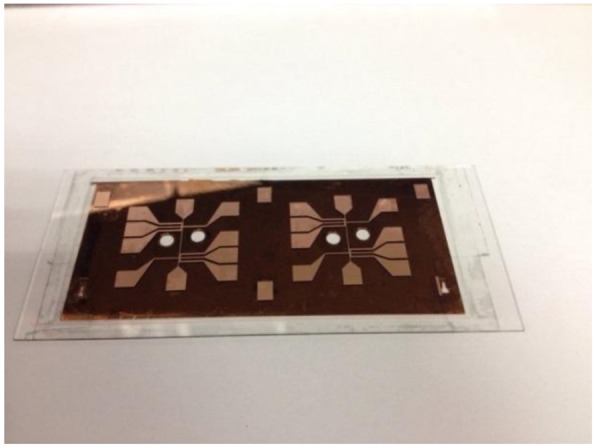
Photograph of a fabricated signal layer transparent atom chip with heat dissipation design on the backside (backside etching).

After HF etching, the residual resist, the masking layer gold and the adhesive layer chromium were removed, and a reactive ion etching (RIE) with the etching gas (O_2_) was applied to clean the surface of the chip. An adhesion layer titanium (20 nm) and a seed layer copper (200 nm) were evaporated on the backside of the chip and the areas of heat dissipation copper blocks were patterned. The heat dissipation copper blocks with a thickness of 100 μm and 200 μm were electroplated on the backside of the chip to conduct the heat, and to enhance the overall mechanical strength of the atom chip. After the electroplating, the front side wires were fabricated with the same process as described in the previous section and finally the titanium and copper layers of light transmitting regions were etched away. Figure 7 shows the photograph of a fabricated signal layer transparent atom chip with heat dissipation design on the backside (backside etching).

### 2.5. Fabrication of the Anti-Reflection Film Coating (AR-Coating)

A laser beam with a wavelength of 780.24 nm is planned to shoot through the transparent atom chip for atomic physics experiments. Thus, in this study an anti-reflection layer was coated on the front and backside of the chip at the laser light transmitting region to improve the laser light transmittance. After fabricating the wires and heat dissipation copper blocks on atom chips, anti-reflection layers of TiO_2_ (85 nm) and SiO_2_ (135 nm) were coated by using an ion-assisted electron gun evaporation system.

## 3. Electrical Test Results

### 3.1. Electrical Resistance Measurements

The width of electroplated copper wires on transparent atom chips are 100 μm and 200 μm, and the heights of all the plated copper wires were set to 20 μm in order to compare the cooling effect caused by different heat dissipation designs. The resistance measurements of the copper wires are used to determine whether the quality of copper wires is consistent during the electroplating process. A four point resistance measurement method is performed to avoid a contact resistance problem and the resistances across the copper wires are measured by a 2002 multimeter (Keithley, Cleveland, OH, USA).

In this study, the electrical resistances of twenty copper wires with a width of 100 μm and 200 μm were measured and the measured results were compared with the calculated theoretical resistance values. The measured, and the calculated theoretical resistances of wire width 100 μm and 200 μm are 0.108 Ω ± 0.00479 Ω and 0.043 Ω ± 0.0031 Ω, and 0.086 Ω and 0.0409 Ω, respectively. This indicates that the measured resistances are higher than the calculated theoretical resistance values. The possible reason may be that the copper wires are deposited by electroplating process and the thickness of the plated copper wire varies along with the unstable current density causing the uneven plated height (errors about 1 μm). In addition, after the copper wire electroplating process is complete, the seed layer of copper film (200 nm) is then removed via the wet etching method. However, due to the wet etching being an isotropic etching, the side walls of the copper wires are also etched while etching the bottom seed layer copper. Both described factors lead to reduce the cross-sectional area of the copper wires, which results in raising the electrical resistance of the wires.

### 3.2. Electrical Current Measurements

To achieve Bose-Einstein Condensation, an appropriate electrical current has to be applied to the wires on the transparent atom chips to generate a sufficient magnetic field to trap atoms. After the calculation of magnetic field, the electroplated copper wires must be capable of carrying several amperes of current according to different atomic physics experiments. However, the temperature of the wires rises continuously while current is running through wires and the electrical resistance of the wires also increase constantly. Then the temperature rising rate of the wires will increase rapidly as well. Due to the above two factors, the accumulation of heat on the wires accelerates until the temperature reaches the melting point of copper, causing the wires to burn out immediately. In order to ensure that the electroplated wires are capable of running several amperes of current through the wires without burnout, the transparent atom chip was directly fixed on an optical bench and an electrical current, ranging from zero to several amperes, was applied through the copper wires on atom chips to check if the maximum burnout current meet our requirements. With the high currents running through the wires, the different heat dissipation designs of this study can also to be examined for their cooling effect. The electrical current measurements are performed only for the wire of width 100 μm on atom chips.

### 3.3. Electrical Current Measurements of Transparent Atom Chips with Heat Dissipation Design on the Front and Back Side (Blind Hole)

**Figure 8.**
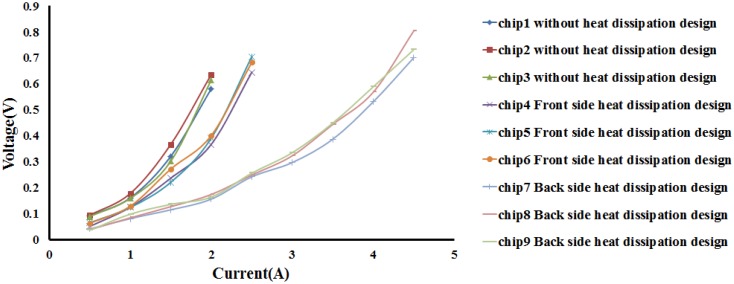
Measured voltage as a function of applied current on atom chips with different heat dissipation design. All the measurements stop at a certain point where the wires burnout.

After the wires were patterned and electroplated on the glass substrate with a thickness of 20 μm, current tests are performed to examine if they could sustain several amperes of current for one minute without burnout. Figure 8 indicates that the measured voltage value on the atom chips without any heat dissipation design increases constantly, which means the electrical resistance in wire is also rising continuously. The wires burned out at the applied electrical current of 2 A due to the glass substrate having a very poor thermal conductivity, causing wires to be unable to dissipate the heat efficiently.

Without any heat dissipation design, the measured maximum burnout current of 2 A is unable to meet the needs of atomic physics. Therefore, in this study several copper blocks were added on the front side of the chip to enhance the heat dissipation. The heat dissipation copper blocks are distributed around the wires and connected to the wire bonding pads. The heat generated from the wires is expected to be effectively conducted to the surrounding copper blocks. Unfortunately, the current measurement results showed that wires were still burned out at a current of 2.5 A and the measured maximum current density is 1.25 × 10^5^ A/cm^2^. A possible reason for this is that the heat in the wires was not rapidly conducted to the copper blocks since the width of the wires is only 100 μm, which causes a huge thermal resistance during the heat conduction process.

Since the copper blocks around the wires on the front side of the chip can't effectively improve the maximum electrical current running through the wires, four tapered blind holes were drilled on the backside of the chip to reduce the thickness of the glass substrate right beneath the wires. This makes the heat from the wires conduct to the backside of the chips more easily. A titanium and copper seed layer was deposited in the blind holes by an E-gun evaporation system, and the blind-holes were then filled by the electroplated copper blocks, which helps to conduct the heat to the backside of the chip.

The electrical current measurement results show that the maximum current running through wires can be raised to 4.5 A and the measured maximum current density is 2.25 × 10^5^ A/cm^2^ as shown in Figure 8, which is a significant improvement compared with the heat dissipation design with the copper blocks on the front side of the chip. This also proves that the thinning of the glass thickness allows the heat generated from the wires to conduct effectively to the backside of the chip. The heat was then further dissipated via the copper blocks in the tapered blind holes so that the wires could withstand a higher electrical current running through.

### 3.4. Electrical Current Measurements of Transparent Atom Chips with Heat Dissipation Design on the Back Side (Backside Etching)

**Figure 9.**
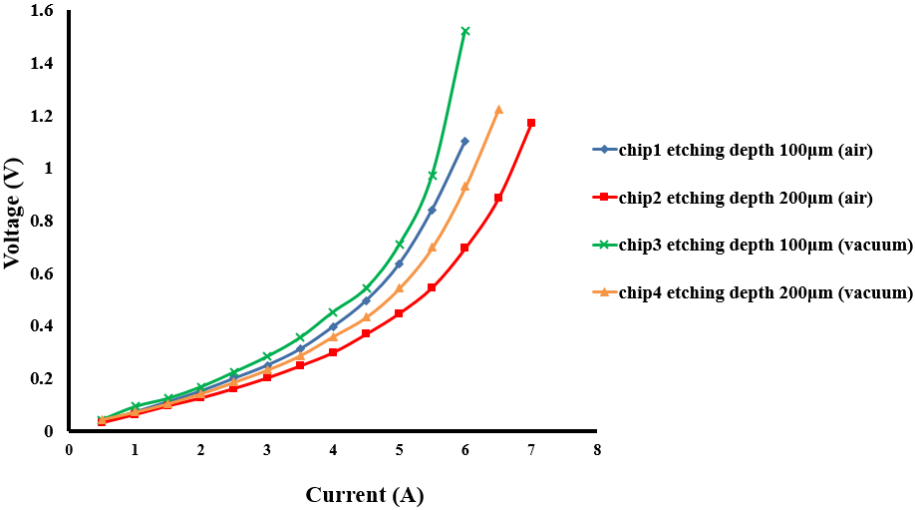
Voltage as a function of applied current from 4 atom chips with backside heat dissipation design (backside etching depth: 100 μm and 200 μm) under different test conditions. “air” and “vacuum” refer to the test being performed at 1 atmosphere and at 1 × 10^−2^ Torr. All the measurements stop at a certain point where the wires burnout.

From the results obtained in the previous section, it is indicated that the heat generated from the wires can be effectively conducted to the backside of the chip with the reduced glass substrate under the wires and thus improving the maximum burnout current. Nevertheless, it is still not high enough to meet the requirement of some specific atomic physics experiments. Therefore, in this study the third heat dissipation design aims to thin the entire glass substrate and fabricate heat dissipation copper blocks with a larger area. A glass substrate with a thickness of 400 μm was used, and the entire surface area on the backside was recessed by HF etching to enhance the heat conduction except the laser light transmitting region. The etching depth is 100 μm and 200 μm so that the glass substrate under the wires is completely thinner and thus heat generated from the wires could be conducted to the backside of the chips more easily. The heat dissipation copper blocks are filled up by the same electroplating process. Two wires with the same width of 100 μm on two different etching depth glass substrates (100 μm and 200 μm) were selected for the electrical current measurements in air as shown in Figure 9. It can be seen from the figure a maximum burnout current of 6 A was achieved when the glass etching depth is 100 μm, and it is further improved to 7 A (maximum current density: 3.5 × 10^5^ A/cm^2^) when the glass etching depth is 200 μm. Both cases improve the maximum burnout current of the wires, which provides a wider application for chip-based atomic physics experiments.

### 3.5. Electrical Current Measurements of Transparent Atom Chips with Heat Dissipation Design on the Back Side (Backside Etching) under Vacuum and in Air

In BEC and atomic physics experiments, an ultra-high vacuum (UHV) environment with a vacuum pressure of 10^−11^ Torr is required and it normally takes a lot of time to reach the UHV level. Thus, the electrical current measurementd under vacuum are important. In order to better simulate the environment in which the wires are expected to perform, several tests are performed under vacuum (1 × 10^−2^ Torr). Two atom chips with different etching depths were placed in a vacuum chamber and the chamber was pumped down to 10^−2^ Torr. A four point contact measurement method is performed to measure the voltage as a function of applied current across the wires. The measured maximum burnout current on two chips with different etching depths were obtained and compared with measurements performed in air. Figure 9 shows the electrical current measurements from the four atom chips with different etching depth of the glass substrate (100 μm and 200 μm) under vacuum (1 × 10^−2^ Torr) and in air. From the current measurement results under two different environments (air and vacuum), it indicates that the measured voltage is proportional to the resistance at the same current value and it can be easily seen that the measured resistance values under vacuum are higher than in air. This means that at the same current, the temperature of the wires under vacuum is higher than in air due to the lack of thermal convection under vacuum, which makes the heat generated from the wires to only dissipate via the thermal conduction to the substrate. Therefore, all the measured electrical resistances under vacuum are higher than in air, which also affects the maximum burnout current running through the wires.

## 4. Conclusions

In this study, a glass substrate is used to fabricate transparent atom chips, so that the laser beam can penetrate directly through the glass substrate. Although the light transmittance of the glass is very good, it is also a substrate with a poor thermal conductivity. This causes the generated heat to be unable to effectively dissipate after a high current runs through the wires which decreases the maximum electrical current that can be applied to the wires. In this study, we first attempted to design and fabricate heat dissipation copper blocks on the front side around the wire patterns of the glass, while retaining a light transmitting region for the laser beam. This is still unable to effectively increase the maximum burnout current, which can only be increased by about 0.5 A via this design. The measured maximum current density is only 1.25 × 10^5^ A/cm^2^. The second heat dissipation design is to drill four tapered blind holes on the backside of the glass substrate right beneath the copper wires to reduce the total thickness of the glass substrate and the heat dissipation copper blocks are then electroplated inside the blind holes. The testing results show that the heat generated by the wires can be effectively conducted to the backside of the substrate for dissipation. A maximum burnout current of only 2 A is obtained without any heat dissipation design on the transparent atom chips and it is increased to 4.5 A (maximum current density: 2.25 × 10^5^ A/cm^2^) after the heat dissipation design with the copper blocks in the blind holes. However, this is still unable to meet certain requirements of some atomic physics experiments, which is running an electrical current of more than 4.5 A through wires. Finally, a large area on the backside of the glass substrate is etched away to reduce the total thickness of the whole atom chips except for the laser light transmitting regions. A heat dissipation copper block is electroplated over the entire backside surface of the atom chips to further increase the maximum burnout current to 7 A (maximum current density: 3.5 × 10^5^ A/cm^2^).
